# Exploratory site survey of Ni availability and accumulation in *Odontarrhena serpyllifolia* across the Bragança massif (Portugal)

**DOI:** 10.1007/s41207-026-01291-w

**Published:** 2026-09-30

**Authors:** Ana R. A. Alves, Eduardo Ferreira da Silva, Luís A. B. Novo

**Affiliations:** 1 GeoBioTec, Department of Geosciences, University of Aveiro, Aveiro, Portugal; 2 School of Natural and Social Sciences, Scotlands Rural College, Edinburgh, Scotland, UK

**Keywords:** Ultramafic soil, Hyperaccumulation, Phytoextraction, Agromining, *Odontarrhena serpyllifolia*, Consumption and Production

## Abstract

Ultramafic soils from the Bragança massif (NE Portugal) harbour the Ni hyperaccumulator species *Odontarrhena serpyllifolia*. The present work investigated the phytomining suitability of this massif and its plant species and examined how different soil properties might hinder or facilitate plant biomass production and Ni accumulation. Plants and soils from four different sites in this massif were collected and analysed. Soils present high Ni concentrations, and all plants exhibited significant Ni accumulation in shoots, however, soil characteristics and plant yields show some variability between sites. Results showed that the highest plant yields were associated with soil pH, greater nutrient availability, particularly P and Na, more favourable Ca: Mg balances, and lower concentrations of soil elements including Mg, Fe, Co and Mn that may contribute to plant stress. Future long-term field trials and testing of soil amendments are key to addressing the full phytomining potential of the Bragança massif and may provide insights applicable to other Mediterranean ultramafic systems hosting Ni hyperaccumulator species.

## Introduction

Metallophytes are plants naturally associated with metal-rich substrates (Baker et al. 2010; Whiting et al. 2002). Through evolutionary adaptation, these plants have acquired physiological and biochemical traits that allow them to tolerate metal concentrations that are harmful to most other species. Hyperaccumulation is one remarkable example of the adaptations of plants to metalliferous environments (Whiting et al. 2002). Hyperaccumulator species concentrate exceptionally elevated concentrations of metals (e.g. As, Cd, Co, Mn, Ni, REEs and Zn) in above-ground tissues under field conditions while maintaining normal growth and reproductive capacity (van der Ent et al. 2013). In general, hyperaccumulators are characterized by tissue metal concentrations that are typically 100 to 1000 times greater than those found in plants growing on non-metalliferous soils and generally exceed concentrations recorded in other metallophytes by at least one order of magnitude (van der Ent et al. 2013). The concentration criteria adopted for hyperaccumulation vary according to the element considered. In dry above-ground tissues, threshold values range from 100 mg kg⁻¹ for Cd, Se and Tl, to 300 mg kg⁻¹ for Co, Cr and Cu, 1000 mg kg⁻¹ for As, Ni and Pb, 3000 mg kg⁻¹ for Zn, and up to 10,000 mg kg⁻¹ for Mn. Hyperaccumulation has so far been recognised in more than 800 plants, and the number continues to increase as new species are described (Fadzil et al. 2024; Paul et al. 2020).

Phytomining involves the cultivation of metal-accumulating plants, primarily hyperaccumulators, for the recovery of economically valuable metals from mineralised or contaminated substrates (Brooks et al. 1998). Growing demand for metals, declining availability of high-grade ore deposits, and environmental concerns associated with conventional mining activities have prompted interest in this phytotechnology (Novo et al. 2017; Reeves et al. 2018; Sheoran et al. 2013). Phytomining is regarded as a sustainable strategy for recovering valuable metals from sub-economic mineral resources that are often uneconomic using conventional mining approaches, and can generate additional environmental benefits (e.g., C sequestration, enhance soil fertility and biodiversity, land restoration) (Echevarria et al. 2015; Kidd et al. 2018; Sheoran et al. 2009). Successful phytomining requires the identification of suitable sites containing both elevated metal concentrations and adapted hyperaccumulator species, followed by plant growth, harvest when appropriate metal accumulation is reached, and biomass processing to generate marketable products (Kidd et al. 2018). Biomass yield and metal concentration are key determinants of phytomining performance because together they control the amount of metal recovered per harvest (harvestable amount) (Novo et al. 2015). Thus, agronomic interventions can be implemented to enhance overall metal recovery (Dang and Li 2022; Nkrumah et al. 2016).

Nickel is widely regarded as one of the most promising targets for phytomining, having received the most attention over the years (Dilshara et al. 2025; Echevarria et al. 2015; Robinson et al. 1997; Rosenkranz et al. 2019). Although global Ni resources are substantial, their geographical distribution is uneven. This metal is widely used in various fields due to its great mechanical strength and ductility, and is crucial for the green energy transition, which led to its recent inclusion as a critical raw material (Nakajima et al. 2014; van Gaalen and Chris Slootweg 2025). Many low-grade Ni resources occur within ultramafic terrains that support a rich diversity of Ni hyperaccumulator species, that also make up approximately 70% of currently recognized hyperaccumulator species (Reeves et al. 2018; Sheoran et al. 2009). Ultramafic formations are distributed globally and collectively account for more than 3% of terrestial land area, with major occurrences in both temperate regions such as the Alps, Türkiye and California, as well as tropical areas including New Caledonia, Cuba and Indonesia (Echevarria 2021; Guillot and Hattori 2013; Kidd et al. 2018).

Ultramafic (serpentine) soils develop through the weathering of ultramafic bedrock. Their mineralogy is dominated by ferromagnesian phases, especially olivine and pyroxene, and they typically contain less than 45% SiO₂ (Kruckeberg 2002). A defining feature of ultramafic soils is the coexistence of nutrient scarcity and metal enrichment. While these substrates often contain substantial quantities of Mg, Fe, Ni, Cr and Co, they are frequently impoverished in essential macro- and micronutrients required for normal plant development, including P, K, Ca, Mo and B (Echevarria 2021; Kidd et al. 2018; Nkrumah et al. 2016). Among the trace metals present, Ni is particularly abundant and may occur at concentrations spanning approximately 700 to 8000 mg kg⁻¹ (Sobczyk et al. 2017). These landscapes are often steep and rocky, producing shallow soils with low organic matter content and restricted water-holding capacity (Kidd et al. 2018). The pedological expression of ultramafic substrates in temperate regions varies considerably, ranging from poorly developed alkaline soils with strong Mg dominance in the exchange complex to more evolved Cambisols displaying near-neutral or slightly acidic conditions (Echevarria 2021; Kidd et al. 2018). These characteristics are harsh for normal plants and make these environments a global hotspot for communities with high rates of endemism (Echevarria 2021; Kidd et al. 2018). The occurrence of Ni hyperaccumulator species is closely linked to these substrates (Kidd et al. 2018; Reeves 2003).

The Mediterranean region constitutes one of the major global centres of diversity for both ultramafic ecosystems and Ni hyperaccumulator flora, with *Odontarrhena* representing one of the most important genera (previously identified as *Alyssum*, family Brassicaceae) (Reeves 2003; Reeves et al. 2018). These ultramafic landscapes are increasingly recognised as important targets for agromining initiatives across southern Europe and the eastern Mediterranean, including sites in locations distributed across Albania, Greece, Italy, Spain and Türkiye (Kidd et al. 2018). Bani et al. (2010, 2013) reported serpentine soil Ni concentrations ranging from 1070 to 3280 mg kg⁻¹ across sites in Albania, Bulgaria and Greece. Among the Ni hyperaccumulators investigated, maximum shoot Ni concentrations reached 20,100 mg kg⁻¹ in *Odontarrhena muralis* (reported as *Alyssum murale*) (Bani et al. 2010). Bani et al. (2013) subsequently reported maximum shoot Ni concentrations of 12,691 mg kg⁻¹ in *O. chalcidica* (reported as *Alyssum markgrafii*) and 9,300 mg kg⁻¹ in *O. muralis* (reported as *A. murale*). *Odontarrhena heldreichii* and *Odontarrhena decipiens* concentrated up to 11,800 mg kg^− 1^ and 17,300 mg kg^− 1^ of Ni, respectively (Bani et al. 2010; Cecchi et al. 2018). In the ultramafic soils of Austria, from four Ni hyperaccumulator species (*O. chalcidica*, *Berkheya coddii*, *Bornmuellera tymphaea* and *Bornmuellera emarginata*), *O. chalcidica* showed the best Ni yields (Hipfinger et al. 2022). *Odontarrhena bertolonii*, the first species ever identified as a Ni hyperaccumulator, from Italy, contains about 10,000 mg kg^− 1^ of Ni in its aboveground dry biomass (Anderson et al. 1999; Minguzzi and Vergnano 1948).

The ultramafic outcrops of the Iberian Peninsula, namely in Portugal and Spain, harbour the hyperaccumulator *O. serpyllifolia* (formerly *Alyssum serpyllifolium*). In Galizia and Andalucía this species registered a Ni accumulation mean in its leaves of 4595 and 2513 mg kg^− 1^, respectively (Pollard et al. 2021). In Portugal, Morais et al. (2015) recorded Ni concentrations in *O. serpyllifolia* of 3405 and 4008 mg kg^− 1^ collected from the Bragança and Morais massif, respectively. Maximum Ni shoot concentrations approached 9500 mg kg^− 1^ (Alves et al. 2019).

Key objectives of this work encompass: (1) analysing the variability of soil characteristics and *O. serpyllifolia* yields from different sites at the Bragança ultramafic massif; (2) evaluating how different soil parameters might influence *O. serpyllifolia* yields; (3) assessing the massif’s potential for Ni phytomining within the broader context of Mediterranean ultramafic environments; and (4) determining which site may be more suitable in the context of phytomining. This study provides a multi-site comparison, within the Bragança massif, between soil physicochemical properties and *O. serpyllifolia* biomass production and Ni accumulation. It identifies key soil properties (e.g., nutrients, metals) and quantifies their co-variation with the Ni hyperaccumulator yields at field scale, providing insight into some edaphic drivers for phytomining. These findings offer a basis for selecting the most suitable sites and agronomic interventions for future phytomining trials.

## Study area and hyperaccumulator species

The presence of ultramafic allochthonous units is a distinctive feature of the geology of north-eastern Portugal (Sequeira et al. 2010). These rocks are scattered in the Bragança and the Morais massifs. Both massifs occur in the Trás-os-Montes subregion, in the Bragança district. The study area, namely the Bragança massif, is located further north near the Portuguese-Spanish border.

**Fig. 1:**
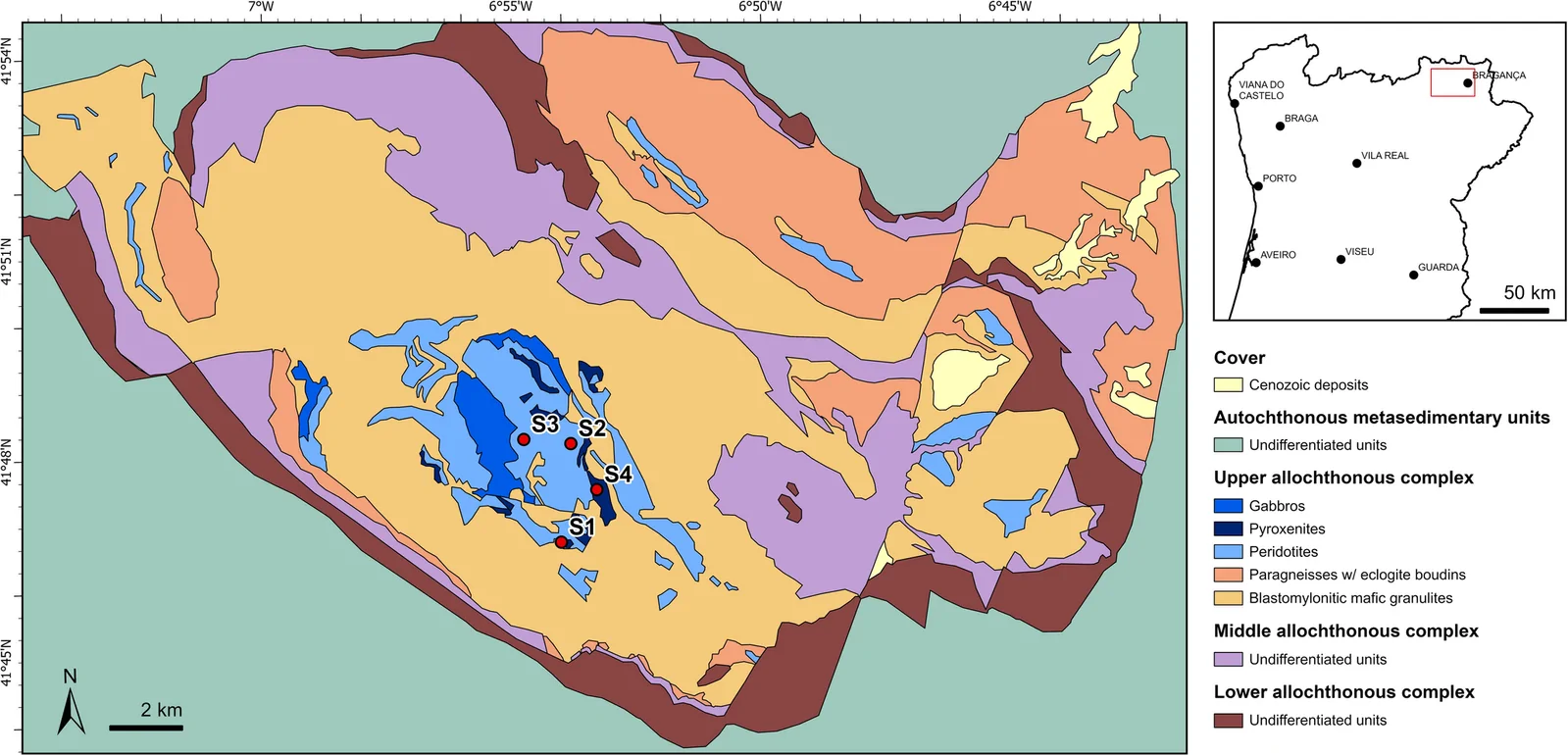
Simplified geological sketch map of the Bragança massif. The red dots labelled S1, S2, S3, and S4 indicate the sampling sites in this study. The red rectangle on the right inset represents the location of the geologically characterized area

Geologically, the Bragança massif (Fig. 1) forms part of the allochthonous domain of the Galicia-Trás-os-Montes Zone (GTMZ) within the Iberian Massif, which tectonically overlaps the Central Iberian Zone (Ribeiro et al. 1979). The allochthonous units recognised in NW Iberia include the Cabo Ortegal, Órdenes, Malpica-Tui, Bragança and Morais complexes (Martínez Catalán et al. 2019). Together with the parautochthon, the complexes form the GTMZ. This zone comprises four tectono-stratigraphic sequences (base to the top): a parautochthonous complex composed mainly of metasediments; a lower allochthonous unit dominated by metasedimentary and metavolcanic lithologies, and gneisses; an ophiolitic complex (intermediate allochthonous unit) represented by rocks such as amphibolite, serpentinized peridotite and gabbro; and an upper allochthonous complex with paragneiss, eclogite, mafic granulite, pyroxenite, and peridotite (Malecki et al. 2024; Marques et al. 1996; Ribeiro et al. 1990).

**Fig. 2:**
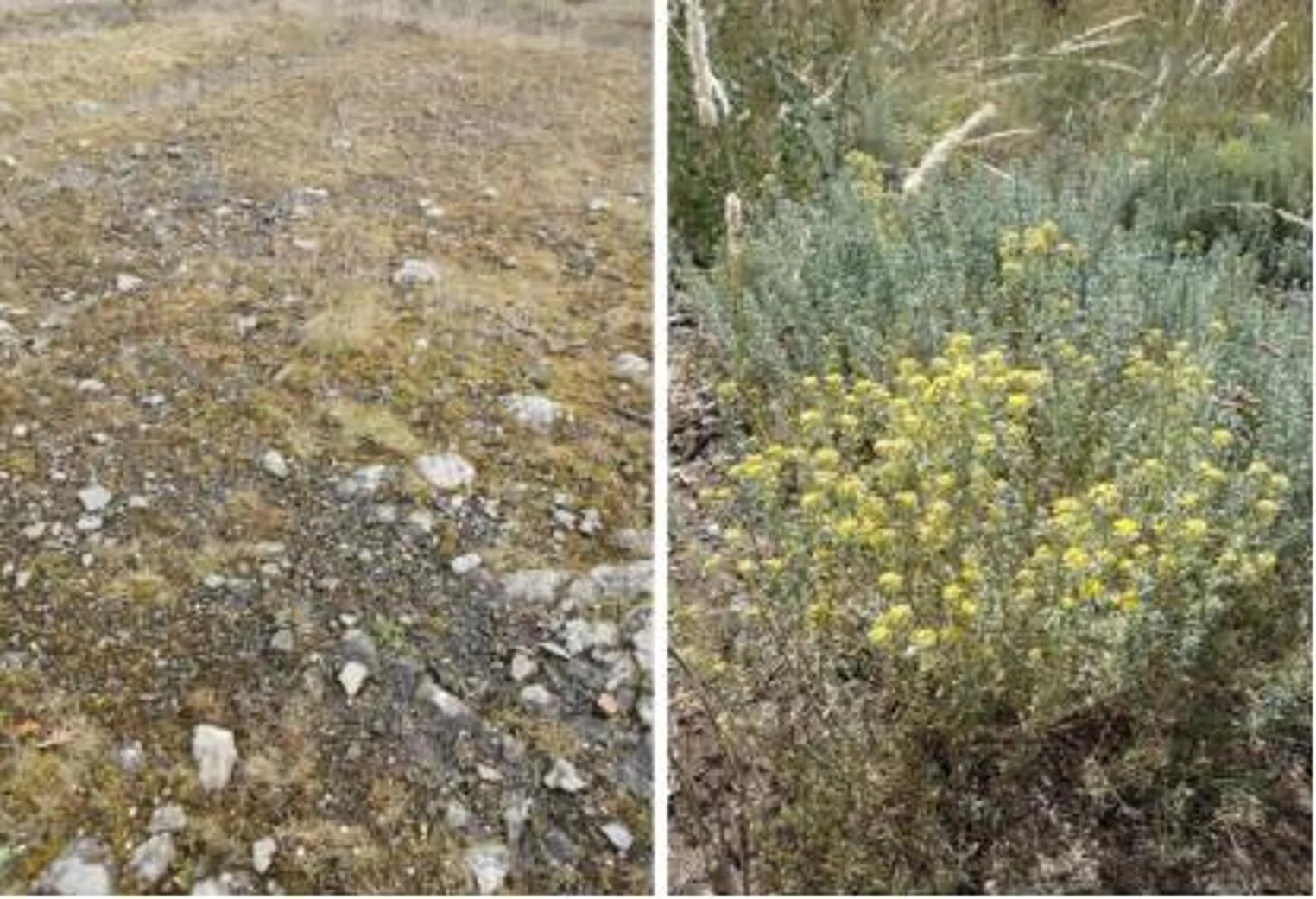
Soil surface at ultramafic sites of the Bragança massif and Ni hyperaccumulator species *Odontarrhena serpyllifolia*

Elevation across the Bragança massif varies from approximately 600 to 1060 m. This region belongs to the cool and relatively wet supra-Mediterranean climatic zone, and it’s characterized by long, cold winters and short hot summers (Morais et al. 2015; Sequeira et al. 2010). The dominant soil type in this massif is a shallow and stony Leptosol (presence of bedrock at 40/50 cm below the surface) (Fig. 2) (Morais et al. 2015). Soil characteristics, namely, toxicity, erosion susceptibility, and slow pedogenesis, limit tree development and overall productivity, thus, shrubs mainly comprise the dominant vegetation cover (Morais et al. 2015).

The plant species *O. serpyllifolia* is distributed throughout the Iberian Peninsula and neighbouring regions, occurring on both limestone and ultramafic substrates (Pollard et al. 2021). Currently, these plant populations that inhabit ultramafic soils and hyperaccumulate Ni are not recognised as taxonomically distinct from those inhabiting other substrates (do not exhibit hyperaccumulation) (Pollard et al. 2021). *O. serpyllifolia* (Fig. 2) from the Bragança and Morais massif was previously named *Alyssum serpyllifolium* ssp. *lusitanicum* T. R. Dudley and P. Silva (= *Alyssum pintodasilvae*) (Dudley 1986).

## Materials and methods

### Sampling

Sampling was carried out at four locations within the Bragança massif, where *O. serpyllifolia* plants and associated soil samples (0–20 cm depth) were collected: (1) 41°46’37.02"N, 6°54’14.62"W; (2) 41°48’5.23"N, 6°54’0.40"W; (3) 41°48’10.10"N, 6°54’56.74"W; (4) 41°47’23.52"N; 6°53’30.65"W (Fig. 1). As observed in Fig. 1, all sites are inserted in the upper allochthonous complex. Geologically, S1, S2 and S3 are in an area with peridotites, whereas site S4 is underlain by pyroxenites. At each site, three individual plants were sampled, together with an associated soil sample (0–20 cm depth) collected from the vicinity of each plant, resulting in three independent plant–soil pairs per site. Plants at a similar developmental stage and overall size were selected to minimise variability. Plants were past the flowering stage, entering senescence. It is important to acknowledge that the three replicate plots may not fully capture the spatial heterogeneity present within each site, particularly in this type of terrain. Sampling was conducted in October 2023, and samples were processed and analysed at the Department of Geosciences, University of Aveiro.

### Soil analysis

Soil samples were dried at room temperature and thoroughly homogenised. Soil texture (classified according to the USDA soil texture classification system) was determined by the weight percentages of sand, silt, and clay separates in the fine-earth fraction (< 2 mm). This was carried out by wet sieving approximately 100 g of soil in distilled water. Samples were mixed with water and washed through sieves with mesh sizes of 2 mm, 1 mm, 500 μm, 250 μm, 125 μm, and 63 μm. The different fractions were collected, oven-dried, and weighed. Finally, granulometric fractions < 63 μm were quantified by gravity sedimentation monitored by X-ray detection using a SediGraph III Plus, using a particle density of 3 g cm⁻³ and calculations based on Stokes’ law and Beer–Lambert extinction principles.

Soil mineralogical content was determined through X-ray diffraction. Approximately 2 g of soil (< 2 mm fraction) was milled to a powder and examined using a Philips X’Pert Pro X-ray diffractometer (40 kV, Cu Kα radiation, λ = 1.5405 Å). XRD patterns were recorded across a 2θ interval (counting step of 0.02˚ per second).

Soil pH and electrical conductivity (EC) from each site were measured in a 1:5 soil-to-water suspension prepared using 5 g of soil (< 2 mm fraction) and 25 mL of distilled water (ISO 11265 1994; ISO/FDIS 10390 2020; Jones 2001). Suspensions were agitated on an end-over-end shaker for 1 h. pH and EC were measured after 1 h and 24 h rest, respectively.

To determine the pseudo-total element concentrations in soils, 0.25 g of each sample, previously milled to a powder, underwent acid digestion using HNO_3_:HCl (3:1, v/v) in a DigiPREP MS digestion block at 95 °C (Ghasemi et al. 2018). Subsequently, the temperature was lowered to 50 °C and, following solvent evaporation, ultra-pure water was added. Elemental concentrations were determined by inductively coupled plasma mass spectrometry (ICP-MS).

Element bioavailability was determined through DTPA extractions (0.005 M DTPA, 0.01 M CaCl_2_, 0.1 M TEA, pH 7.3) using 20 mL of DTPA solution for every 10 g of soil (< 2 mm fraction) (FAO 2022). The suspension was agitated on an end-over-end shaker at 30 rpm for 2 h. Later, the resulting extract was passed through a fine-porosity filter paper (Whatman No. 42) and analysed by ICP-MS. The method applied is standard for Cu, Fe, Mn, Ni and Zn.

### Plant analysis

Plant material was rinsed thoroughly with tap water and subsequently with distilled water. Root and shoot tissues were separated and dried in a forced-air oven at 50 °C for 48 h. Dry biomass was recorded, and roots and shoots were ground into a powder. Later, 0.25 g of plant samples underwent acid digestion using HNO_3_:H_2_O_2_ (3:2, *v/v*) in a digestion plate (DigiPREP MS) at 85 °C. Later, the temperature was reduced to 50 °C. Following solvent evaporation, ultra-pure water was added. Plant elemental concentrations were quantified by ICP-MS.

Plant capacity for Ni uptake from soil was assessed, and their proficiency in translocating Ni from roots to shoots as well using bioconcentration (BCF) and translocation (TF) factors, respectively (Eqs. 1 and 2) (Padmavathiamma and Li 2007; Zhuang et al. 2007). The amount of Ni recovered per plant, termed the harvestable amount (HA), was calculated following Eq. 3.

1 $$BCF=\frac{shoot\:Ni\:concentration}{soil\:Ni\:concentration}$$

2 $$TF=\frac{shoot\:Ni\:concentration}{root\:Ni\:concentration}$$

3 $$HA=biomass\: \times \:shoot\:Ni\:concentration$$

### Statistical analysis

Replicate measurements (*n* = 3) were used for all statistical evaluations. Site effects were assessed by one-way ANOVA, following verification of variance structure using Levene’s, Brown-Forsythe’s and Welch’s tests. Mean separation was performed with Fisher’s LSD test under homogeneous variance conditions and with Dunnett’s T3 test when variance heterogeneity was detected. All calculations were carried out in IBM SPSS Statistics 30.0^®^.

Associations between measured parameters were investigated using Pearson’s correlation coefficients generated in R (v4.5.2)^®^. Correlation analyses were based on the 12 individual paired plant–soil observations (three per site). Because numerous pairwise statistical tests were performed, p-values were adjusted using the Benjamini-Hochberg method to control the expected false discovery rate.

## Results

### Soil physical characteristics and mineralogy

Soil surface from the study sites in the Bragança massif exhibits brown to red colours, has a considerable number of small stones and gravel, and its texture is classified as sandy loam. Mineralogically, it is composed primarily of clay minerals, serpentine, amphiboles, olivine, pyroxene, and plagioclase.

### Soil physicochemical properties and element concentrations

Table 1 presents some soil physicochemical properties and element pseudo-total concentrations from the different locations in the Bragança massif. Soil pH means values range from 6.4 to 7.5, and they differ significantly among sites, with S2 exhibiting the highest pH, followed by S4, S3, and finally S1. Regarding EC, S3 and S2 show significantly higher and lower values, respectively, whereas S1 and S4 are not substantially different.

**Table 1:** Soil physicochemical parameters and element pseudo-total concentrations (mg kg^− 1^) from different sites of the Bragança massif

	S1	S2	S3	S4
pH	6.42 ± 0.06^d^	7.50 ± 0.15^a^	6.70 ± 0.14^c^	7.01 ± 0.08^b^
EC (µS cm^− 1^)	28.00 ± 1.58^b^	19.08 ± 1.64^c^	46.20 ± 2.07^a^	29.12 ± 0.48^b^
*Element pseudo-total concentrations* (mg kg^− 1^)				
Ca	9189 ± 658^a^	1685 ± 48^d^	8297 ± 41^b^	4387 ± 78^c^
Mg	45,212 ± 632^c^	129,013 ± 1371^a^	44,682 ± 730^c^	47,939 ± 95^b^
Ca: Mg	0.20 ± 0.01^a^	0.01 ± 0.00^d^	0.19 ± 0.00^b^	0.09 ± 0.08^c^
P	1085 ± 13^b^	449 ± 8^d^	1682 ± 48^a^	508 ± 0^c^
K	578 ± 35^c^	< 200^d^	751 ± 7^a^	672 ± 13^b^
B	< 20^*^	< 20^*^	< 20^*^	< 20^*^
Cu	193.4 ± 1.2^a^	25.6 ± 0.0^d^	105.7 ± 0.9^c^	152.9 ± 0.7^b^
Fe	42,890 ± 692^c^	68,558 ± 807^b^	67,847 ± 684^b^	87,706 ± 875^a^
Mn	510 ± 6^d^	1120 ± 15^b^	1092 ± 12^c^	1472 ± 4^a^
Mo	< 0.2^*^	< 0.2^*^	< 0.2^*^	< 0.2^*^
Ni	862 ± 2^d^	2494 ± 5^a^	972 ± 8^c^	2100 ± 16^b^
Zn	44.1 ± 0.9^c^	33.3 ± 0.6^d^	63.3 ± 1.0^a^	52.5 ± 0.2^b^
Cr	492 ± 20^b^	285 ± 4^d^	338 ± 3^c^	587 ± 16^a^
Co	66.2 ± 0.4^d^	130.3 ± 0.4^b^	90.8 ± 0.5^c^	145.7 ± 2.1^a^
Na	1378 ± 138^a^	< 200	655 ± 4^b^	281 ± 26^c^

Values represent mean ± standard deviation (*n* = 3). Within each parameter, different lowercase letters denote significant differences among sites (*p* < 0.05)

*Limit of quantification

As expected from ultramafic soils, all sites presented high concentrations of the dominant elements Mg and Fe, together with the trace metals Co, Cr and Ni (Hossner 2008; Kabata-Pendias 2011). The quantities of these metals vary across the four locations (Table 1). Magnesium has significantly higher concentrations at S2 than at S4. S1 and S3 show the lowest Mg concentrations, with no statistically significant difference between them. Regarding Fe, S4 exhibits the significantly highest concentration. S2 and S3 (not statistically different from each other) have significantly greater Fe values than S1. Mean Ni pseudo-total recorded soil Ni concentrations varied between 862 and 2494 mg kg^− 1^. S2 showed significantly higher Ni quantities than the other sites, followed by S4, S3, and finally S1. Chromium concentrations are higher in S4, followed by S1, S3, and last, S2. Cobalt concentrations are highest in S4, followed by S2, S3, and S1.

The Ca: Mg ratios were very low, and all sites showed statistically significant differences between them (Bani et al. 2013). Mean ratios of 0.01, 0.09, 0.19, and 0.20 were obtained for S2, S4, S3, and S1, respectively. Regarding plant macronutrients, P values were significantly higher in S3, followed by S1, S4, and lastly S2. S3 and S2 also exhibited the highest and lowest K concentrations, respectively. S4 has a significantly higher quantity of this nutrient than S1. Table 1 also presents concentrations of the micronutrients Mn, Zn, Cu, B and Mo, together with the beneficial element Na, across the four sampling locations.

### Soil element bioavailability

Table 2 summarizes the bioavailable concentrations of selected elements in soils collected from the four sampling locations within the Bragança massif. Bioavailability is the fraction of a chemical element available for uptake by organisms. For the most part, the values obtained for each chemical element vary significantly between the sampling sites. Regarding Ni, bioavailability is highest at S4, followed by S1, S2, and S3.

**Table 2:** Bioavailable element concentrations (mg kg^− 1^) in soils from different sites of the Bragança massif

	S1	S2	S3	S4
Mg	539 ± 7^b^	873 ± 3^a^	408 ± 2^c^	412 ± 4^c^
P	1.86 ± 0.12^a^	< 0.5*	0.82 ± 0.01^c^	1.01 ± 0.01^b^
K	11.2 ± 0.3^c^	3.2 ± 0.1^d^	18.6 ± 0.1^b^	36.4 ± 0.3^a^
Cu	7.48 ± 0.05^b^	1.25 ± 0.02^d^	2.58 ± 0.04^c^	7.82 ± 0.12^a^
Fe	51.5 ± 0.5^a^	30.8 ± 0.1^b^	22.8 ± 0.2^d^	23.9 ± 0.7^c^
Mn	25.0 ± 0.5^d^	43.5 ± 0.4^c^	53.8 ± 0.6^b^	93.7 ± 1.5^a^
Ni	39.8 ± 0.4^b^	34.7 ± 0.2^c^	23.9 ± 0.5^d^	64.8 ± 1.4^a^
Zn	0.310 ± 0.000^c^	0.170 ± 0.000^d^	0.423 ± 0.012^b^	0.447 ± 0.006^a^
Co	1.82 ± 0.05^c^	1.5 ± 0.01^d^	1.91 ± 0.04^b^	2.51 ± 0.03^a^
Cr	0.027 ± 0.002^a^	0.006 ± 0.000^c^	0.005 ± 0.000^c^	0.020 ± 0.001^b^

Values represent mean ± standard deviation (*n* = 3). Within each parameter, different lowercase letters denote significant differences among sites (*p* < 0.05)

*Limit of quantification

### Plant biomass and Ni uptake

**Fig. 3:**
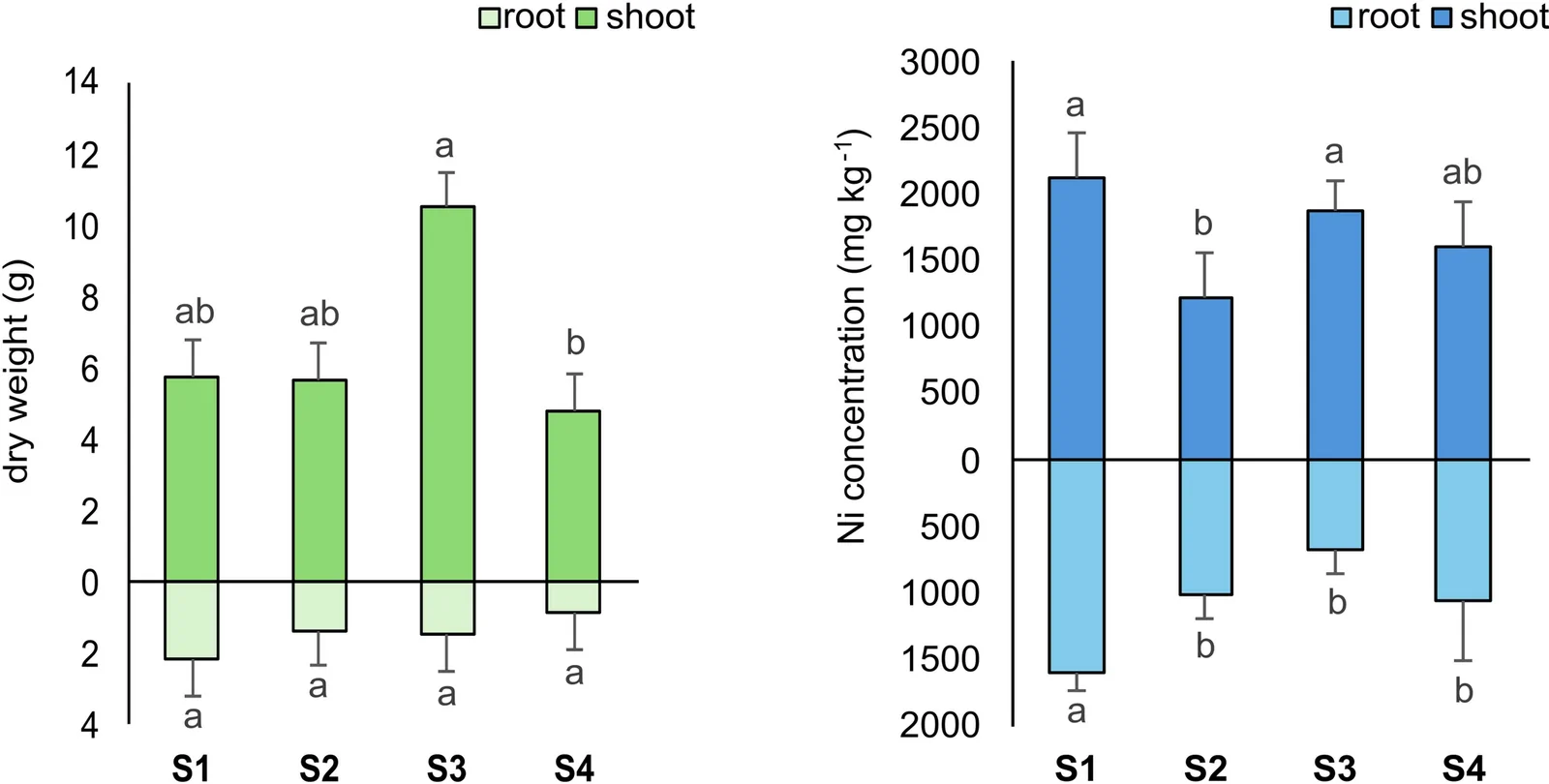
Dry weights (g) and Ni concentrations (mg kg^− 1^) of *Odontarrhena serpyllifolia* roots and shoots from different sites of the Bragança massif. Values represent mean ± standard deviation (*n* = 3). Within each parameter, different lowercase letters denote significant differences among sites (*p* < 0.05)

Mean dry biomass and Ni concentrations measured in roots and shoots of *O. serpyllifolia* from plants collected in October at the different sites are presented in Fig. 3. Root dry weights did not differ significantly; nevertheless, the mean was highest in S1 (2.2 g), followed by S3, S2, and S4. Regarding plant shoots, weights were higher in S3 (10.5 g) and were significantly different from those in S4. Weights recorded from S1 and S2 are not significantly different from each other or from those at the other sites.

**Fig. 4:**
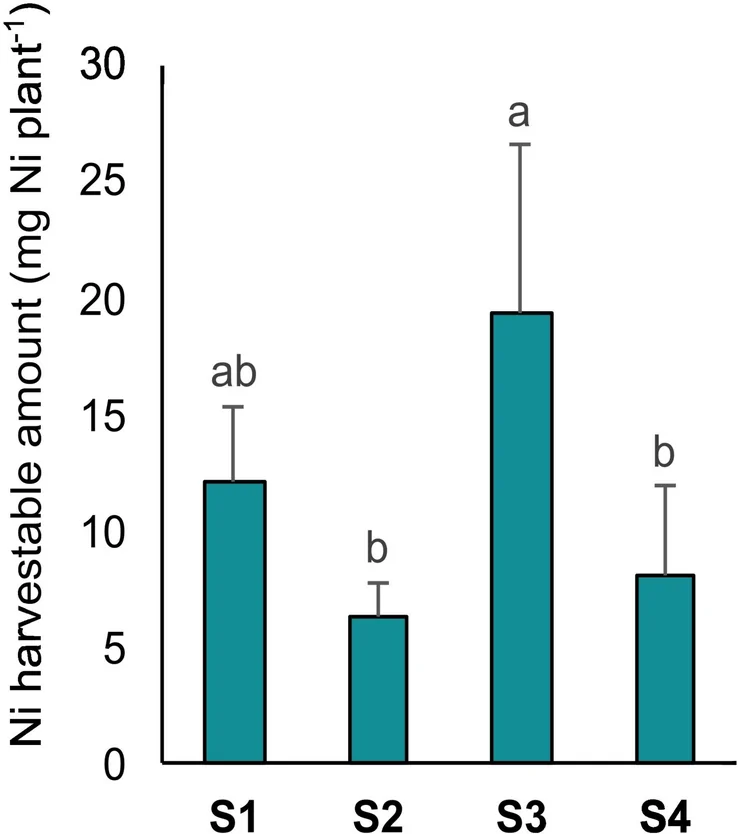
*Odontarrhena serpyllifolia* nickel harvestable amounts from different sites of the Bragança massif. Values represent mean ± standard deviation (*n* = 3). Within each parameter, different lowercase letters denote significant differences among sites (*p* < 0.05)

Nickel concentrations in roots are significantly greater in S1 (1602 mg kg^− 1^) compared with the other locations. No significant differences were detected in root Ni concentrations between the remaining locations. Regarding shoot Ni concentrations, S1 (2133 mg kg^− 1^) and S3 (1870 mg kg^− 1^) accumulated significantly more than S2 (1217 mg kg^− 1^). Shoot Ni concentrations at S4 (1614 mg kg⁻¹) did not differ significantly from those recorded at the remaining sites. The maximum shoot Ni concentration observed was 2504 mg kg⁻¹, at S1. In accordance with shoot biomass and Ni concentrations, Ni HA (Fig. 4) was greater in S3 (19.3 mg Ni plant⁻¹), followed by S1 (12.2 mg Ni plant⁻¹), S4 (8.1 mg Ni plant⁻¹), and lastly S2 (6.3 mg Ni plant⁻¹).

### Plant efficiency to uptake Ni

**Fig. 5:**
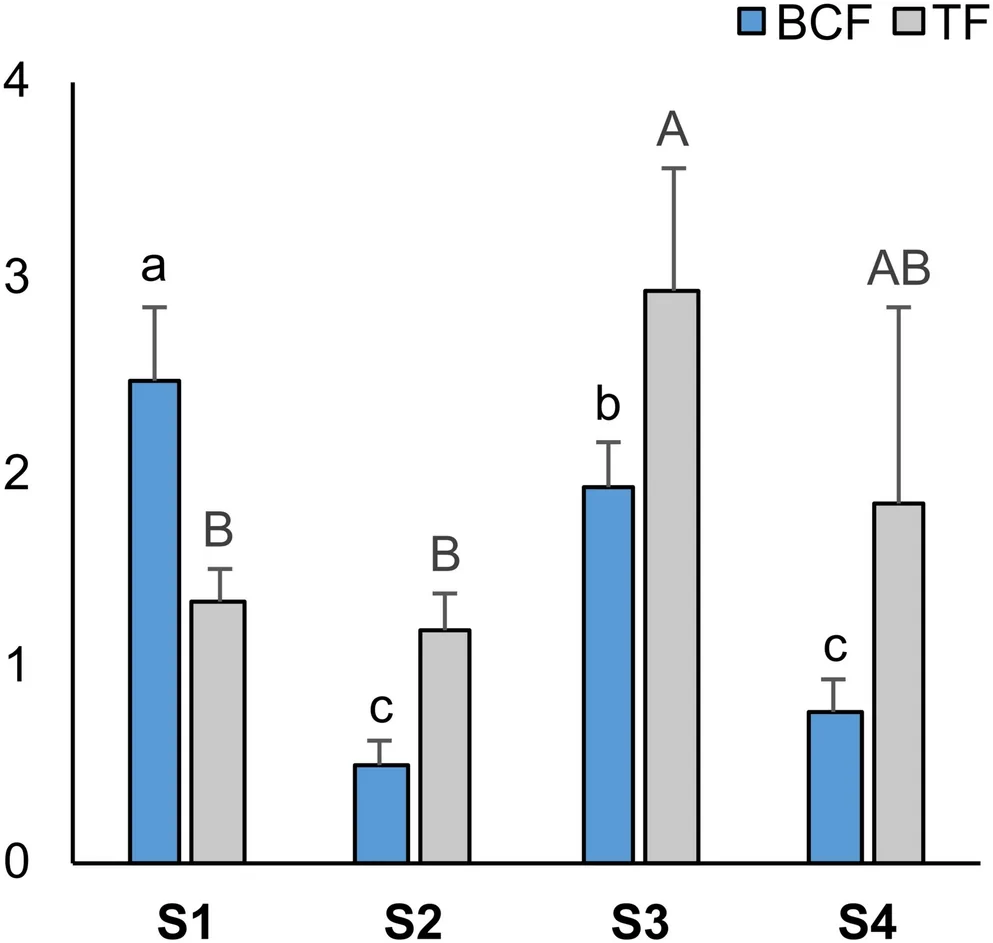
Bioconcentration factor (BCF) and translocation factor (TF) of *Odontarrhena serpyllifolia* from different sites of the Bragança massif. Values represent mean ± standard deviation (*n* = 3). Within each parameter, different lowercase letters denote significant differences among sites (*p* < 0.05)

*Odontarrhena serpyllifolia* efficiency to uptake Ni from soil (BCF) and translocate this metal from roots to shoots (TF) is presented in Fig. 5. Nickel uptake from soil is significantly higher in S1, followed by S3. Sites 2 and 4 exhibited the lowest BCF value. Plants are more efficient at translocating Ni from root to shoot in S3, followed by S4, S1, and finally S2; however, S3 does not differ statistically from S4. The TF is not statistically different between S1, S2, and S4.

### Element concentrations in plant shoots

Table 3 shows some element concentrations in *O. serpyllifolia* shoots from the different sites. No significant differences were observed among locations for shoot concentrations of Mg, Ca, Ca: Mg, Co or Mn.

**Table 3:** *Odontarrhena serpyllifolia* shoot element concentrations (mg kg^− 1^) from different sites of the Bragança massif

	S1	S2	S3	S4
Ca	13,245 ± 5422a	12,579 ± 10197a	19,353 ± 6014a	16,627 ± 6025a
Mg	5603 ± 1847a	6332 ± 3839a	6677 ± 1852a	9714 ± 2740a
Ca: Mg	2.3 ± 0.2a	1.9 ± 0.5a	2.9 ± 0.7a	1.9 ± 1.1a
Cu	7.1 ± 5.4a	0.6 ± 0.3b	1.2 ± 0.9b	0.3 ± 0.2b
Fe	2497 ± 1121a	595 ± 278b	1076 ± 508ab	1502 ± 1101ab
Mn	51 ± 17a	41 ± 19a	58 ± 4a	59 ± 28a
Zn	33 ± 19a	6 ± 3b	9 ± 7b	5 ± 1b
Co	8 ± 4a	3 ± 1a	7 ± 2a	4 ± 2a
Cr	34 ± 17a	7 ± 4b	10 ± 5b	19 ± 15ab
Na	127 ± 62a	45 ± 14b	79 ± 7ab	60 ± 27b

Values represent mean ± standard deviation (*n* = 3). Within each parameter, different lowercase letters denote significant differences among sites (*p* < 0.05)

## Discussion

Soil characteristics, including those presented in Tables 1 and 2, can influence plant growth, biomass production, and Ni uptake by hyperaccumulators, and they vary among sampling sites in the Bragança massif (Dilshara et al. 2025). This is also true for *O. serpyllifolia* biomass production and Ni accumulation, which is reflected by the different Ni harvestable amounts obtained at each site - although not always significantly different. Therefore, evaluating site characteristics and the factors controlling Ni uptake is essential for understanding differences in phytomining performance.

The soil mineralogy from the Bragança massif reveals the geology of the units present in the study area (Fig. 1). The soils were characterised by pH values ranging from slightly acidic to slightly alkaline, elevated concentrations of Mg, Fe, Ni, Cr and Co, low Ca: Mg ratios, and comparatively low levels of P and K. These characteristics are typical of ultramafic soils from other temperate and Mediterranean regions, including serpentine systems reported from Albania, Greece, Italy, Spain, and Türkiye (Alves et al. 2019; Bani et al. 2024; Echevarria 2021; Hossner 2008; Kabata-Pendias 2011; Morais et al. 2015). The principal constraints affecting plant growth on ultramafic soils are generally considered to be low Ca: Mg ratios, elevated concentrations of potentially toxic metals, particularly Ni and Mg, and limited P availability (Brady et al. 2005; Ghasemi and Ghaderian 2009; Kazakou et al. 2008).

**Fig. 6:**
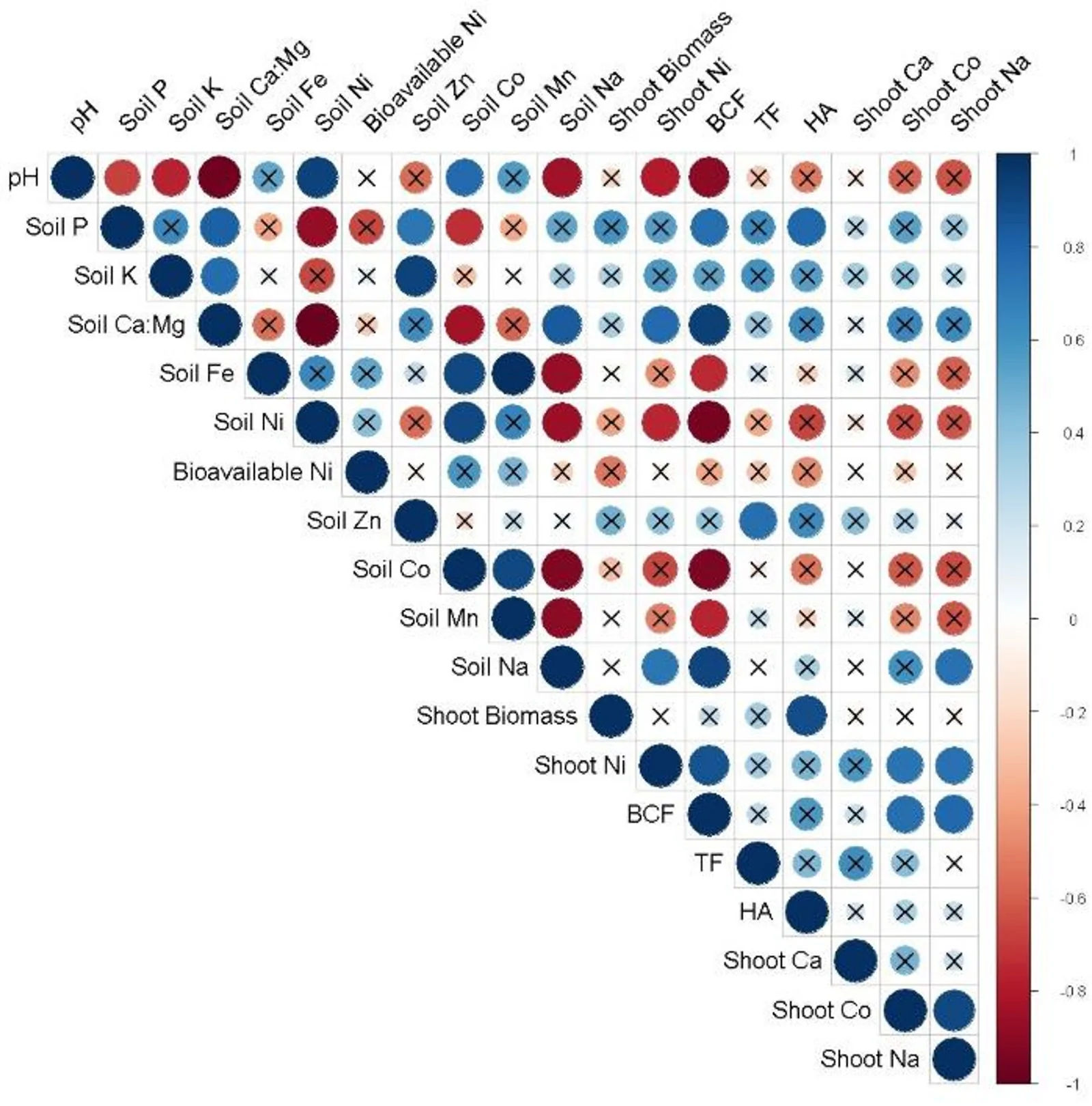
Pearson correlations between the studied parameters at different sites. Correlations are significant at *p* < 0.05, except on cells marked with an X

Figure 6 shows correlations between the studied parameters. Soil pH influences metal solubility and mobility in soil (Sheoran et al. 2009). This parameter is widely recognised as a major factor controlling metal bioavailability and is known to enhance Ni uptake by plants, with several studies reporting enhanced Ni uptake under lower pH conditions (Bani et al. 2024; Dilshara et al. 2025; Mühlbachová et al. 2011). Some other factors affecting metal availability include soil metal composition, organic matter, clay abundance, redox conditions and root exudation processes (Antić-Mladenović et al. 2011; Chardot et al. 2007; Nkrumah et al. 2016). In this study, absolute DTPA-extractable Ni concentrations did not show a simple relationship with soil pH across the four sites. However, these concentrations need to be considered in the context of the markedly different pseudo-total Ni concentrations among sites. For example, although S1 contained substantially less pseudo-total Ni than S2 (862 vs. 2494 mg kg⁻¹), its DTPA-extractable Ni concentration was higher (39.8 vs. 34.7 mg kg⁻¹), consistent with the considerably lower pH of S1 (6.42 vs. 7.50). Conversely, the highest DTPA-extractable Ni concentration was observed at S4 (64.8 mg kg⁻¹), where the pseudo-total Ni concentration was also considerably higher than at S1 (2100 vs. 862 mg kg⁻¹). Thus, the observed Ni availability likely reflects the combined influence of the size of the soil Ni pool and soil physicochemical conditions, including pH.

Soil pH was positively correlated with soil Ni concentrations but negatively correlated with Ni concentrations measured in plant shoots. This means that soils with lower pH displayed lower Ni pseudo total concentrations and greater Ni concentrations in plant shoots. In this case, S1 has the lower pH and greater Ni accumulation by *O. serpyllifolia*, followed by S3, S4, and finally S2. This is also true for the BCF, indicating that plants uptake more Ni from soil overall. This corroborates the findings of previous studies, although it is inconsistent with the Ni bioavailability results. Different Ni pools (total, pseudo-total and bioavailable) may respond differently to soil physicochemical conditions. Moreover, plant metal uptake is complex and depends not only on bioavailable concentrations but also on factors such as soil density, diffusion processes and root development (Uchimiya et al. 2020; Podar and Maathuis 2022). Nickel hyperaccumulators possess highly effective root-mediated mechanisms capable of significantly reshaping Ni pools and local chemical equilibria over time. For instance, roots can deplete bioavailable Ni pools, which may subsequently be replenished from less labile pools (Centofanti et al. 2012). The apparent incongruence between DTPA-extractable Ni and shoot Ni concentrations may therefore also reflect dynamic soil–plant interactions and site-specific soil characteristics. Given the limited number of sampling sites and replicates, these relationships should nevertheless be interpreted with caution.

Plant nutrients in soil, such as P and K, are known to generally increase plant establishment and biomass production, including in Ni hyperaccumulator species (Álvarez-López et al. 2016; Nkrumah et al. 2016). This may, to some extent, explain the variability in plant weights across locations, with the HA being positively correlated with pseudo-total soil P concentrations.

In plant crops, the macronutrient Ca is known to reduce Ni uptake and phytotoxicity (Chaney et al. 2008). Very low Ca: Mg ratios (< 1) are widely recognised as a major cause of reduced fertility in ultramafic soils, and it was hypothesized that the high Mg concentrations are antagonistic to plant Ca uptake (Kazakou et al. 2008; Marschner 2002). In this work, positive associations were observed between Ca: Mg ratios, shoot Ni concentrations and BCF values. Evidence from nutrient solution experiments indicates that greater Ca availability promotes Ni accumulation in shoots, increases plant growth, and facilitates the transfer of Ni from roots to aerial tissues (Chaney et al. 2008). The authors suggested that these responses result from higher Ca ratios alleviating Ni toxicity and thereby enhancing phytoextraction efficiency.

No correlation was found between Ni and Ca concentrations in *O. serpyllifolia* shoots. However, this was previously observed for other Ni hyperaccumulator plants (van der Ent and Mulligan 2015). Shoot Ca concentrations measured in this study (mean: 15451 mg kg^− 1^) were greater than Mg concentrations (mean: 7082 mg kg^− 1^), meaning *O. serpyllifolia* absorbed more Ca relative to Mg (Table 3). Other studies also show that species from serpentine soils are better at promoting a greater Ca absorption relative to Mg than plants from normal soils, which substantially reduces Mg and Ni toxicity and is a crucial evolutionary adaptation to these environments (Bani et al. 2014; O’Dell et al. 2006; Walker et al. 1955). Similar variability in Ni accumulation and biomass production has been reported across Mediterranean ultramafic environments, where local soil chemistry, Ca: Mg ratios, nutrient limitations, and trace metal interactions strongly influence agromining performance of *Odontarrhena* species and other Ni hyperaccumulators (Bani et al. 2010, 2014; Kidd et al. 2018). These findings further highlight the importance of site-specific evaluations when assessing phytomining opportunities in Mediterranean serpentine environments.

Higher quantities of Fe in soil resulted in a lower BCF. Studies propose that hyperaccumulator plants absorb Ni through low-affinity transport pathways that are largely associated with Zn and Fe transporters (Deng et al. 2018; Van der Pas and Ingle 2019). Both elements have previously been reported to interfere with Ni accumulation in shoot tissues (Deng et al. 2018; Taylor and Macnair 2006; Van der Pas and Ingle 2019). In contrast, a study from 2018 finds no evidence that these metals hinder Ni accumulation (Mohseni et al. 2018). Our results show that soil Zn concentrations were positively correlated with the translocation of Ni from roots to shoots. Higher Fe and Zn concentrations in plant shoots did not negatively affect shoot Ni concentrations (Table 3).

Besides the challenges posed by nutrient deficiencies and unfavourable Ca ratios, elevated levels of Cr, Co and Mn may act as additional stressors in ultramafic ecosystems, potentially affecting plant growth and performance (Kazakou et al. 2008). In this case, a negative association was found between Co in soil and *O. serpyllifolia* BCF. The same was true for Mn. A study observed that among the *Odontarrhena* species Ni hyperaccumulation is associated with the potential to accumulate Co at a similar degree (Homer et al. 1991). In our work, a correlation was also found between Ni quantities and Co in plant shoots.

Regarding Na, positive correlations were observed between soil Na concentrations, shoot Ni concentrations and BCF values. There is also an association between Ni shoot and Na shoot concentrations. Sodium is not considered essential for most plant species but may provide benefits under certain conditions, particularly where K availability is limited (Maathuis 2014).

Biomass production and Ni accumulation values recorded for *O. serpyllifolia* in this study are lower than those previously reported for plants inhabiting the Bragança massif (Morais et al. 2015). This is largely explained by the timing of sampling, as specimens were collected in October, well after the peak flowering and maximum biomass stage, when above-ground tissues were already entering senescence. Sampling at this late developmental stage nevertheless provides information on Ni retention in mature and partially senescent tissues and represents a conservative assessment of harvestable Ni under suboptimal harvest conditions. Consequently, the Ni HA reported here is likely to underestimate the species’ phytomining potential at peak biomass. Furthermore, plant density was not determined in this field survey, precluding a reliable extrapolation of Ni yields per unit area. Such an extrapolation could also misrepresent the phytomining potential of *O. serpyllifolia*, as the present study assessed naturally occurring plants without agronomic optimisation and after peak biomass production. Dedicated field trials should therefore assess *O. serpyllifolia* under controlled planting densities and agronomic management, with harvesting at peak biomass, to establish realistic Ni yields per hectare.

Despite reduced biomass at the time of collection, plants from all sites accumulated elevated Ni concentrations in their shoots, which, when combined with remaining above-ground biomass, resulted in measurable and site-dependent HA values. In particular, plants from sites S1 and S3 yielded the highest HA of Ni, reflecting the combined effect of comparatively higher shoot Ni concentrations and greater biomass rather than differences in uptake or translocation capacity alone, which, although validated for both sites (Fig. 5), are inherent traits of Ni hyperaccumulators. These patterns suggest that *O. serpyllifolia* retains substantial amounts of Ni even beyond peak growth, and that sites S1 and S3 represent the most promising locations for further targeted investigations. As discussed, this enhanced performance is likely linked to more favourable soil conditions at these sites, including slightly lower pH, improved nutrient availability (P, K, Ca and Na), more favourable Ca: Mg ratios, and reduced concentrations of potentially phytotoxic elements such as Mg, Fe, Co and Mn. The incorporation of soil amendments that would provide the abovementioned nutrients, adjust Ca: Mg ratios, and mitigate heavy metal stress, would be interesting for future trials in Bragança and similar Mediterranean ultramafic systems.

## Conclusion

Soil properties, and various other factors, drive plant biomass production and Ni accumulation in Mediterranean ultramafic systems and thereby influence the effectiveness of the phytomining process. Thus, a thorough understanding of these parameters is essential for future implementation of this phytotechnology. In this study, we were able to assess that slightly lower pH, higher quantities of P, Ca and Na, and lower concentrations of the metals Mg, Fe, Co and Mn are linked to the greater Ni yields obtained from *O. serpyllifolia* in the Bragança massif. Furthermore, sites S1 and S3 showed the greatest potential for further phytomining investigations, as indicated by the higher plant Ni harvestable amounts recorded at these sites. Appropriate agronomic practices and soil amendments could further increase Ni hyperaccumulator yields and improve the viability of the phytomining process. In this case, the selection of amendments that might add nutrients, moderately reduce soil pH, and mitigate the stress caused by high metal concentrations could lead to better results. Future testing and long-term field trials in the Bragança massif with *O. serpyllifolia* are key to determining the full potential of this species and may contribute to the development of agromining strategies for comparable Mediterranean ultramafic environments.

## Data Availability

Data will be made available on request.
